# Supplementary material for: Creation of a biological sensorimotor interface for bionic reconstruction
Source: Nat Commun. 2024 Jun 24;15:5337. doi: 10.1038/s41467-024-49580-8 (PMC11196281; doi:10.1038/s41467-024-49580-8)
Supplement: Supplementary file 2 — Reporting Summary [file 41467_2024_49580_MOESM2_ESM.pdf]

nature portfolio

Corresponding author(s): Christopher Festin

Last updated by author(s): May 23, 2024

Reporting Summary

Nature Portfolio wishes to improve the reproducibility of the work that we publish. This form provides structure for consistency and transparency in reporting. For further information on Nature Portfolio policies, see our Editorial Policies and the Editorial Policy Checklist.

Statistics

For all statistical analyses, confirm that the following items are present in the figure legend, table legend, main text, or Methods section.

n/a	Confirmed	
☐	☒	The exact sample size (*n*) for each experimental group/condition, given as a discrete number and unit of measurement
☐	☒	A statement on whether measurements were taken from distinct samples or whether the same sample was measured repeatedly
☐	☒	The statistical test(s) used AND whether they are one- or two-sided<br>*Only common tests should be described solely by name; describe more complex techniques in the Methods section.*
☒	☐	A description of all covariates tested
☐	☒	A description of any assumptions or corrections, such as tests of normality and adjustment for multiple comparisons
☐	☒	A full description of the statistical parameters including central tendency (e.g. means) or other basic estimates (e.g. regression coefficient) AND variation (e.g. standard deviation) or associated estimates of uncertainty (e.g. confidence intervals)
☐	☒	For null hypothesis testing, the test statistic (e.g. F, t, r) with confidence intervals, effect sizes, degrees of freedom and P value noted<br>*Give P values as exact values whenever suitable.*
☒	☐	For Bayesian analysis, information on the choice of priors and Markov chain Monte Carlo settings
☒	☐	For hierarchical and complex designs, identification of the appropriate level for tests and full reporting of outcomes
☒	☐	Estimates of effect sizes (e.g. Cohen's *d*, Pearson's *r*), indicating how they were calculated

Our web collection on statistics for biologists contains articles on many of the points above.

Software and code

Policy information about availability of computer code

Data collection	No software was used.
Data analysis	Prism (version 9.0.2; GraphPad Software, USA), SPSS (version 29.0.0.0; IBM, USA), MatLab (R2010a, The MathWorks Inc., USA)

For manuscripts utilizing custom algorithms or software that are central to the research but not yet described in published literature, software must be made available to editors and reviewers. We strongly encourage code deposition in a community repository (e.g. GitHub). See the Nature Portfolio guidelines for submitting code & software for further information.

Data

Policy information about availability of data

All manuscripts must include a data availability statement. This statement should provide the following information, where applicable:

- Accession codes, unique identifiers, or web links for publicly available datasets
- A description of any restrictions on data availability
- For clinical datasets or third party data, please ensure that the statement adheres to our policy

The data supporting the findings of this study are available within this paper and its supplemental material.

Research involving human participants, their data, or biological material

Policy information about studies with human participants or human data. See also policy information about sex, gender (identity/presentation), and sexual orientation and race, ethnicity and racism.

Reporting on sex and gender	This information was not collected.
Reporting on race, ethnicity, or other socially relevant groupings	Groupings were not used.
Population characteristics	This information was not collected.
Recruitment	No human participants were recruited.
Ethics oversight	This study protocol did not involve human participants.

Note that full information on the approval of the study protocol must also be provided in the manuscript.

Field-specific reporting

Please select the one below that is the best fit for your research. If you are not sure, read the appropriate sections before making your selection.

☒ Life sciences ☐ Behavioural & social sciences ☐ Ecological, evolutionary & environmental sciences

For a reference copy of the document with all sections, see nature.com/documents/nr-reporting-summary-flat.pdf

Life sciences study design

All studies must disclose on these points even when the disclosure is negative.

Sample size	This study was experimental in nature and it was not possible to calculate a sample size. Sample sizes were determined based on relevant, previous studies in this field, our group's experience in animal research and advice from a statistician of our institute.
Data exclusions	No data was exlcuded.
Replication	All attempts at replication were successful (thick cut staining n=8, other stainings n=12/n=13, Retrograde labeling n=8, ENG n=13)
Randomization	Randomization was not relevant to this study as it was an experimental animal model investigating surgical feasibility.
Blinding	As there was no group allocation, blinding was not necessary.

Reporting for specific materials, systems and methods

We require information from authors about some types of materials, experimental systems and methods used in many studies. Here, indicate whether each material, system or method listed is relevant to your study. If you are not sure if a list item applies to your research, read the appropriate section before selecting a response.

Materials & experimental systems

n/a	Involved in the study	
☐	☒	Antibodies
☒	☐	Eukaryotic cell lines
☒	☐	Palaeontology and archaeology
☐	☒	Animals and other organisms
☒	☐	Clinical data
☒	☐	Dual use research of concern
☒	☐	Plants

Methods

n/a	Involved in the study	
☒	☐	ChIP-seq
☒	☐	Flow cytometry
☒	☐	MRI-based neuroimaging

Antibodies

Antibodies used	chicken anti-Neurofilament 1:2500 (Merck Millipore, USA; AB5539 LOT 3996248), goat anti-ChAT 1:100 (Merck Millipore, USA; AB144P LOT 3926290), Dako Omnis rabbit anti-S100 1:1 (Dako, Agilent Technologies, USA; GA504 LOT 41631194), mouse anti-MBP 1:500 (Santa Cruz Biotechnology, USA; SC-271524 LOT K1922)

Validation	The antibodies were validated by their respective manufacturers (including literature cited by the manufacturer) or have been used in previous studies cited in the manuscript.

Animals and other research organisms

Policy information about studies involving animals; ARRIVE guidelines recommended for reporting animal research, and Sex and Gender in Research

Laboratory animals	Male Sprague Dawley rats (Charles River Laboratories, Germany) aged 8-10 weeks
Wild animals	This study did not involve wild animals.
Reporting on sex	Sex was considered regarding the housing of the animals and were not relevant for any other part of this study.
Field-collected samples	This study did not involve field-collected samples.
Ethics oversight	The project was approved by the institutional review board and the Austrian Federal Ministry of Education, Science and Research (BMBWF 2020-0.171.173)

Note that full information on the approval of the study protocol must also be provided in the manuscript.

Plants

Seed stocks	Plant specimens were not collected.
Novel plant genotypes	No plant genotypes were used.
Authentication	Plants were not used.
