# Supplementary material for: Creation of a biological sensorimotor interface for bionic reconstruction
Source: Nat Commun. 2024 Jun 24;15:5337. doi: 10.1038/s41467-024-49580-8 (PMC11196281; doi:10.1038/s41467-024-49580-8)
Supplement: Supplementary file 3 — Supplementary Material [file 41467_2024_49580_MOESM3_ESM.pdf]

Supplemental Material

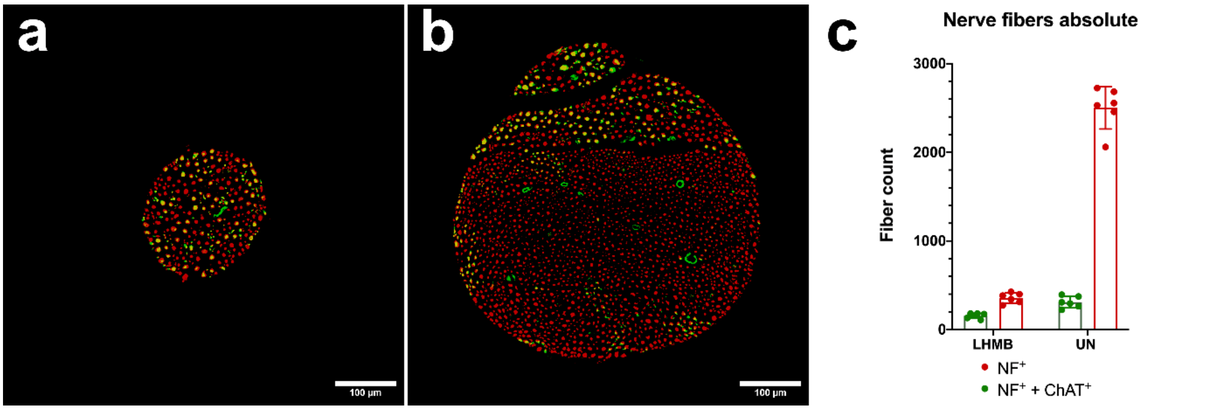

Supplemental Figure S1: Analysis of the involved peripheral nerves. (n=6, biological replicates) *Anti-NF/ChAT staining of the contralateral LHMB (**A**) and UN (**B**). NF (red) is a general neural marker, while ChAT (green) specifically highlights cholinergic structures. Thus, the overlay of the two (yellow) indicates efferent axons, while red axons are of afferent nature. (**C**) The respective peripheral nerve fiber counts (presented as mean and standard deviation). (NF = neurofilament, ChAT = choline acetyltransferase, LHMB = long head's muscle branch, UN = ulnar nerve)*

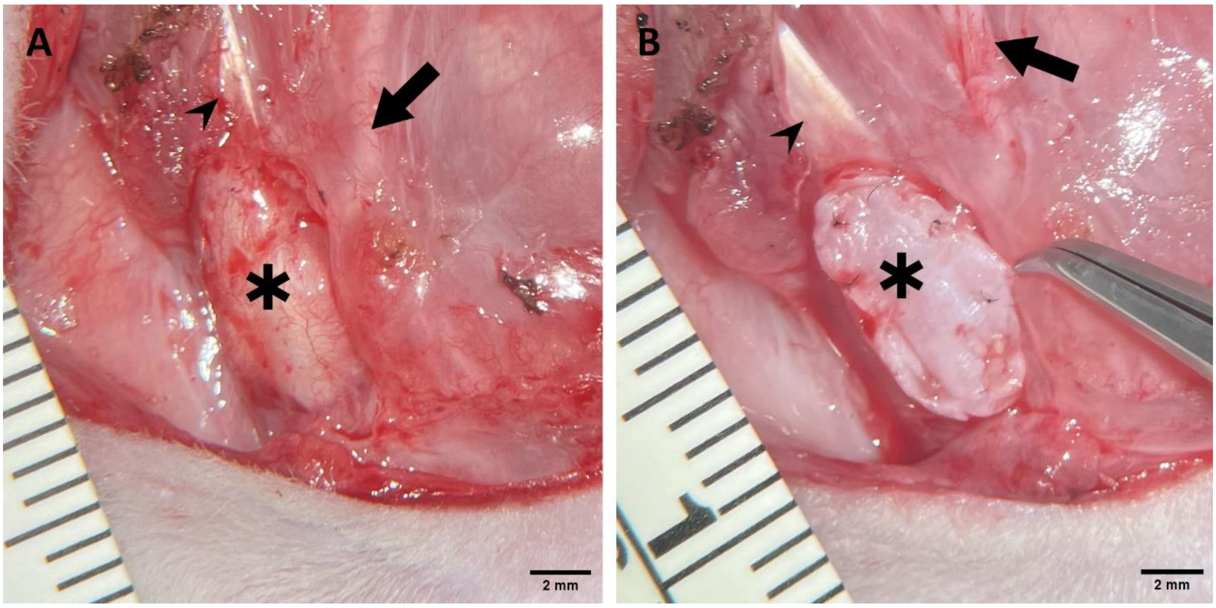

Supplemental Figure S2: The interface after a 12-week follow-up period. *(**A**) An epidermoid cyst can be seen on top of the dermal graft (*). (**B**) After cyst removal, the graft (*) appears fully revascularized and engrafted on top of the bicep's long head. The small arrows mark the bicep's proximal tendon, while the larger arrows point at the transferred ulnar nerve.*

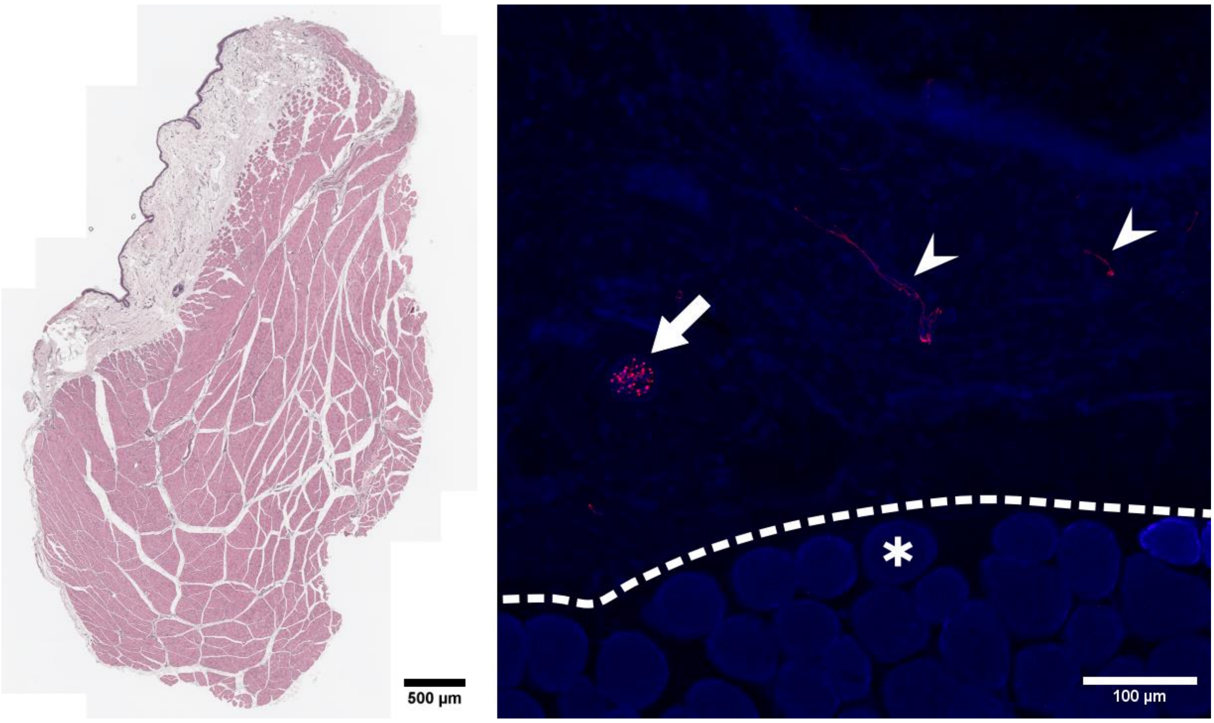

Supplemental Figure S3: Morphological analysis of a muscle-skin sample. *(**Left**) Hematoxylin and eosin stained cross section. The skin is fully engrafted and the muscle shows an intact architecture, thus suggesting successful motor reinnervation. (**Right**) Anti-neurofilament stained cross section of the musculocutaneous junction. The dashed line separates the muscle fibers (*) from the dermal graft. Bundles of axons (large arrow) as well as individual fibers (small arrows) can be seen throughout the graft, suggesting sensory reinnervation.*

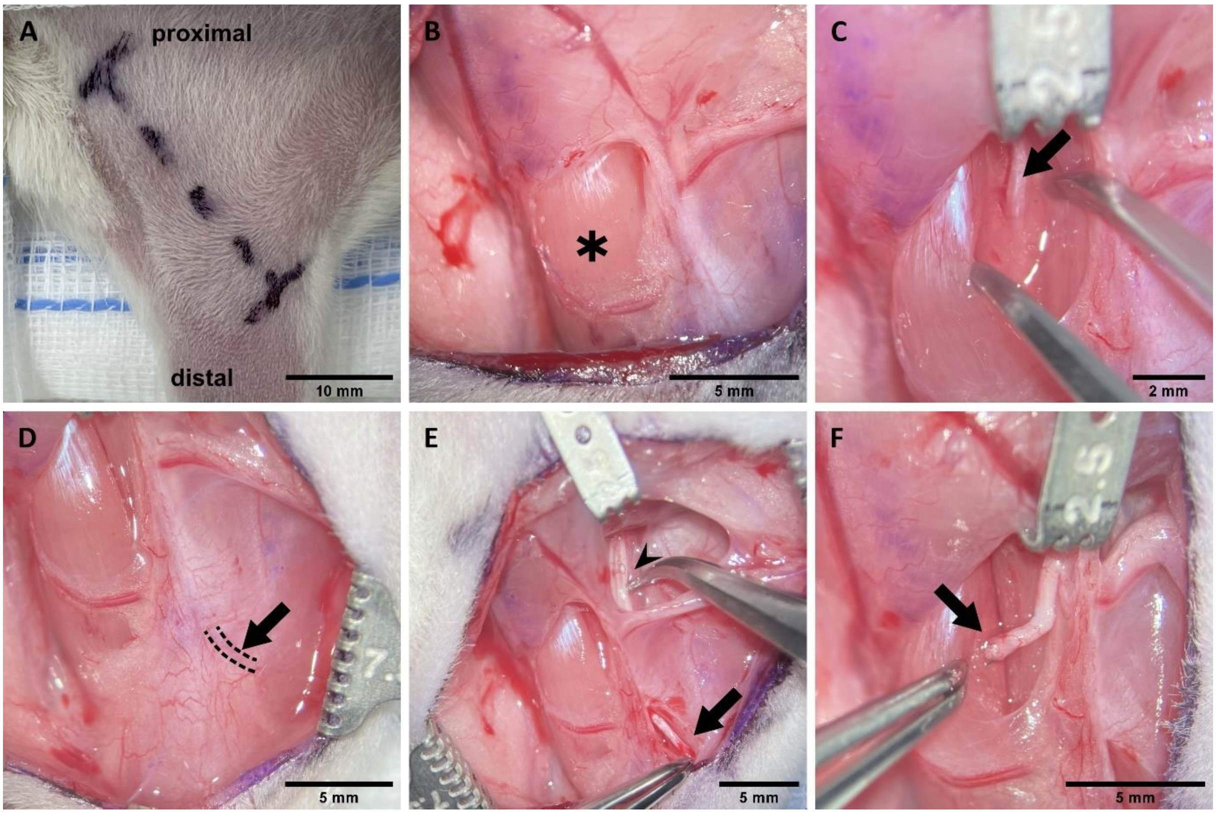

Supplemental Figure S4: Overview of the surgical procedure. *(**A**) View of the abducted upper extremity. An incision is made between the acromion and the medial epicondyle. (**B**) Dissection of the long head of the biceps (*) and removal of its fascia. (**C**) Separation of the muscle's two heads, dissection of the long head's muscle branch (arrow) followed by its transection further proximal. (**D**) A faintly visible, accompanying vein (arrow) guides the ulnar nerve's dissection. (**E**) The nerve is exposed via a small window (large arrow), dissected, and transected before entering the cubital tunnel. The ulnar nerve is then located further proximal in the medial bicipital groove (small arrow) to mobilize it for the nerve transfer. (**F**) The ulnar nerve is transferred to the long head of the biceps (arrow).*

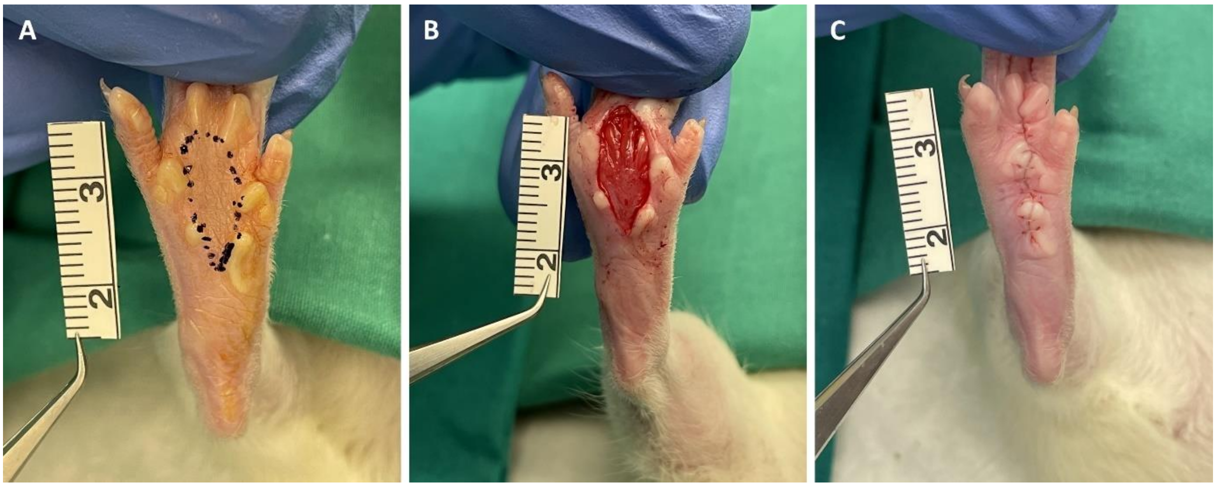

Supplemental Figure S5: Harvesting of the glabrous dermal skin graft. *(**A**) An approximately 1.5x0.5cm graft is marked between the walking pads. (**B, C**) The resulting defect can be easily closed primarily using 6-0 absorbable sutures.*

Sample	only NF+	NF/ChAT+	Total
LHMB 1	180	132	312
LHMB 2	203	181	384
LHMB 3	167	175	342
LHMB 4	164	110	274
LHMB 5	220	180	400
LHMB 6	271	155	426
UN 1	2306	224	2530
UN 2	2247	309	2556
UN 3	2331	394	2725
UN 4	2195	263	2458
UN 5	2310	374	2684
UN 6	1765	295	2060

Supplemental Table S1: Axon counts for the peripheral nerves. *(NF = neurofilament, ChAT = choline acetyltransferase, LHMB = long head's muscle branch, UN = ulnar nerve)*

Sample	FB	RB	DL
C8 1	820	69	55
C8 3	832	235	197
C8 4	993	149	135
C8 5	980	292	235
C8 6	813	59	47
C8 7	923	52	44
C8 8	1088	93	86
C8 9	511	110	106
T1 1	742	115	84
T1 2	869	226	132
T1 3	1134	335	254
T1 4	760	81	66
T1 5	752	130	98
T1 6	1084	108	76
T1 7	1348	132	114
T1 8	1532	138	123
T1 9	1840	487	403
SC 1	177	0	0
SC 2	176	1	0
SC 3	140	4	2
SC 4	253	0	0
SC 5	200	0	0
SC 6	114	0	0
SC 7	203	0	0
SC 8	206	0	0
SC 9	115	0	0

Supplemental Table S2: Cell counts for the dorsal root ganglia C8/T1 and spinal cords. *(FB = fast blue, RB = Red Retrobeads™, DL = double labeled, SC = spinal cord)*

Animal	Monofilament 2 g	Monofilament 4 g	Monofilament 6 g	Monofilament 8 g	Monofilament 10 g	Monofilament 15 g
	[µV]	[µV]	[µV]	[µV]	[µV]	[µV]
E01	0.14	0.18	0.17	0.23	**0.55**	**0.62**
E02	0.37	0.40	0.34	**0.61**	**0.68**	-
E03	0.53	**0.74**	0.64	**0.59**	0.59	-
E04	0.13	0.20	0.22	0.24	**0.40**	**0.39**
E05	0.16	0.11	0.17	0.18	0.14	0.11
E06	0.42	**0.63**	**0.57**	**0.81**	**0.98**	**0.84**
E07	0.15	0.16	0.16	0.28	**0.40**	**0.62**
E08	0.13	0.21	0.28	0.40	0.33	**0.56**
E09	0.22	0.20	0.26	0.15	0.31	0.26
E10	0.19	0.22	0.40	0.33	**0.49**	**0.59**
E11	0.17	0.18	0.22	0.36	0.41	**0.65**
E12	0.21	**0.55**	**0.52**	**0.82**	**0.79**	**1.16**
E13	**0.60**	**0.83**	**0.62**	**1.05**	**1.14**	**2.03**
C01	0.18	0.17	0.17	0.26	0.25	**0.54**
C02	0.22	**0.58**	**0.51**	**0.73**	**0.69**	**0.86**
C03	0.22	0.43	0.29	**0.46**	**0.51**	**0.90**
C04	0.32	0.27	**0.33**	**0.49**	**0.57**	**0.61**
C05	0.12	0.08	0.12	0.13	0.13	**0.35**
C06	0.36	**0.90**	0.58	**1.23**	**1.03**	**1.72**
C07	0.29	0.45	0.42	**0.79**	**1.10**	**1.45**
C08	0.40	**0.88**	0.58	**1.23**	**2.15**	**2.79**
C09	0.21	0.38	**0.50**	**0.66**	**0.74**	**1.29**
C10	0.46	0.62	0.43	**0.77**	**1.08**	**1.13**

Supplemental Table S3: Electroneurography results following mechanical stimulation with different monofilaments. *Overview of the recorded maximum amplitudes for the experimental group with skin grafting (E) and the control group (C). Clearly identifiable signals are bold and underlined. 2 data points are missing due to an operating error during the recording sessions.*

Animals	Vibration superficial	Vibration 1 mm	Undulating vibration	Consecutive tendon pulling	Constant tendon pulling	Perineural vibration
	[µV]	[µV]	[µV]	[µV]	[µV]	[µV]
E01	**1.12**	**2.86**	**3.15**	-	-	
E02	**1.81**	**2.64**	**2.52**	-	-	
E03	**3.20**	**5.43**	**2.78**	-	-	
E04	**1.80**	**2.16**	**1.96**	-	-	
E05	0.15	**0.72**	**0.38**	-	-	
E06	**2.34**	**4.19**	**2.03**	-	-	
E07	**1.29**	**2.10**	**1.39**	-	-	
E08	**0.91**	**1.69**	**1.06**	-	-	
E09	0.33	**1.21**	**0.61**	-	-	
E10	**1.74**	**3.05**	**2.60**	-	-	
E11	**1.05**	**4.15**	**1.13**	-	-	
E12	**1.73**	**3.51**	**1.59**	-	-	
E13	**2.43**	**3.84**	**1.20**	-	-	
C01	**0.98**	**2.60**	**1.39**	**1.30**	**1.10**	0.26
C02	**2.65**	**4.36**	-	**0.85**	**0.78**	0.17
C03	**0.74**	**2.59**	**1.64**	**1.61**	**1.01**	0.20
C04	**0.99**	**3.02**	**2.42**	**1.24**	**1.00**	0.26
C05	**0.53**	**1.30**	**0.70**	**0.31**	0.22	0.19
C06	**4.12**	**1.60**	**12.51**	**5.39**	**6.21**	0.66
C07	**1.14**	**6.14**	**8.33**	**4.23**	**3.80**	0.64
C08	**2.23**	**9.57**	-	**3.37**	**3.00**	0.10
C09	**3.15**	**7.20**	**5.05**	**2.24**	**1.71**	0.25
C10	**1.59**	**9.47**	**4.42**	**2.57**	**2.72**	0.44

Supplemental Table S4: Electroneurography results following vibration and pulling of the tendon. *Overview of the recorded maximum amplitudes for the experimental group with skin grafting (E) and the control group (C). Mechanical stimulation via manipulation of the tendon and perineural vibration was only performed in the control group. Clearly identifiable signals are bold and underlined. 2 data points are missing due to an operating error during the recording sessions.*

Sample	n1 [µm]	n2 [µm]	n3 [µm]	n4 [µm]	n5 [µm]
C8 1	5.06	5.12	5.31	4.87	5.12
C8 3	6.05	5.77	5.42	4.59	6.13
C8 4	5.77	5.89	5.41	6.24	4.79
C8 5	5.32	5.78	4.89	5.5	6.24
C8 6	4.78	6.17	5.89	5.88	4.25
C8 7	5.6	5.46	5.11	5	5.11
C8 8	5	5.64	5.73	5.83	3.84
C8 9	6.12	5.67	5.76	5	6.61
T1 1	6.11	5.33	3.36	5.81	6.64
T1 2	4.99	4.97	5.5	6.25	5.66
T1 3	5.21	5.93	5.15	5.06	6.32
T1 4	4.46	5.24	4.74	4.51	4.28
T1 5	6.99	4.54	4.9	5.13	6.78
T1 6	5.09	4.78	6.21	5.36	2.58
T1 7	4.41	5.37	4.39	5.36	5.31
T1 8	5.2	5.4	5.72	5.02	4.96
T1 9	4.31	4.73	5.34	4.6	5.1
SC 1	5.14	5.51	5.16	5.99	3.73
SC 2	5.38	4.8	4.37	5.46	4.68
SC 3	4.46	5.56	5.44	5.33	4.45
SC 4	6.89	6.95	6.06	7.18	6.42
SC 5	4.88	6.18	6.44	5.77	4.63
SC 6	4.17	3.9	2.45	2.88	5.17
SC 7	5.68	6.31	4.91	4.85	6.26
SC 8	4.09	6.8	5.6	7.1	6.29
SC 9	5.65	5.71	6.45	5.46	6.16

Supplemental Table S5: Nucleoli diameters. *Five diameters were measured per sample. (SC = spinal cord)*
